# Monocyte Chemotactic Proteins Mediate the Effects of Hyperglycemia in Chondrocytes: In Vitro Studies

**DOI:** 10.3390/life12060836

**Published:** 2022-06-03

**Authors:** Adam Quincey, Subburaman Mohan, Bouchra Edderkaoui

**Affiliations:** 1 Musculoskeletal Disease Center, Research Service, VA Loma Linda Healthcare Systems, Loma Linda, CA 92357, USA; adam.quincey@va.gov (A.Q.); subburaman.mohan@va.gov (S.M.); 2 Department of Medicine, Loma Linda University, Loma Linda, CA 92354, USA; 3 Department of Biochemistry, Loma Linda University, Loma Linda, CA 92354, USA; 4 Department of Orthopedic Surgery, Loma Linda University, Loma Linda, CA 92354, USA

**Keywords:** chemokines, chondrocytes, high glucose

## Abstract

Chemokines are secreted by a large variety of cells. They are involved in controlling cell trafficking, maturation, and differentiation. However, the specific responses and effects of chemokines on specific skeletal cell types under high glucose conditions have not been investigated. Chondrocytes play an important role in osteoarthritis and fracture healing. Delayed fracture healing is one of the major health complications caused by diabetes, so the goal of this study was to evaluate the response of several chemokines to high glucose conditions in chondrocyte cells and analyze their role in the catabolic effect of hyperglycemia. ATDC5 chondrocytes were cultured in normal and high glucose media, and mRNA expression levels of several chemokines and chondrocyte differentiation markers were quantified. Bindarit, a specific inhibitor of monocyte chemotactic proteins (MCPs), was used to determine the role of MCPs in mediating the effects of high glucose conditions in chondrocyte cells. High glucose treatment upregulated the expression of three *Mcps*, as well as the expression of matrix metalloproteinase 13 (*Mmp13*) and Osteocalcin (*Oc*). Furthermore, bindarit treatment downregulated *Mmp13* and *Oc* but upregulated Collagen 2 (*Col2)* mRNA levels in chondrocytes treated with high glucose. Moreover, treatment of chondrocytes with ascorbic acid reduced the effect of high glucose conditions on the expression of chemokines and *Mmps*. These data together suggest that MCPs mediate the catabolic effect of high glucose in chondrocytes.

## 1. Introduction

Chemokines are implicated in many diseases such as diabetes [1,2], osteoarthritis (OA) [3,4,5], and osteoporosis [6]. Chemokines function through interactions with their receptors. Their primary role is to induce inflammation through chemotaxis of monocytes, lymphocytes, neutrophils, eosinophils, basophils, natural killer cells, dendritic cells, and endothelial cells [7,8,9,10,11], but they also have direct effects on many cells [12,13]. Chemokines are key regulators of wound healing and bone fracture healing processes [14], but uncontrolled expression of chemokines correlates with delayed wound and fracture healing [14,15]. Among MCP family members, monocyte chemotactic protein-1 (MCP1), also known as CCL2, is the most studied chemokine in osteoarthritis (OA), diabetes and bone fracture healing fields [2,14,16,17,18,19]. Previous studies have demonstrated that MCP1 is involved in bone remodeling and plays a crucial role in early post-fracture healing phase [20,21,22]. However, prolonged expression of *Mcp1* was observed in diabetic patients that suffered from bone fragility and delayed fracture healing [23,24]. Moreover, recent studies showed that alteration of MCP1 interaction with its specific receptor, C-C chemokine receptor type 2 (CCR2), reduced macrophage infiltration to injured knees and protected the knees from cartilage damage in mice with OA [4], while in vitro studies showed that stimulation of chondrocytes with MCP1 enhanced chondrocyte hypertrophy [25]. OA is a multifactorial disease, based in part on inflammation and aberrant differentiation of articular chondrocytes (ACs). Under normal conditions, ACs remain in a resting state and refrain from proliferation or terminal differentiation. However, in OA conditions, the cells enter a cascade of proliferation and hypertrophic differentiation stages accompanied by markers of differentiation such as collagen 10 [26], and MMP-13 [27], with subsequent apoptotic death [28] and mineralization of articular cartilage [29]. Furthermore, previous studies [30] have reported that high glucose (HG) induced the expression of *Mcp1* and toll like receptors (*Tlr*) 4 and 2, and inhibition of TLR4, but not TLR2, reduced the effect of HG on the expression of *Mcp1* and nuclear NF-κB p65 protein in human peritoneal mesothelial cells [30], suggesting that HG induces *Mcp1* expression partly through TLR4/NF-κB signaling pathway. These data showed that MCP1 plays an important role in several inflammatory diseases. However, less is known on the role of other MCP family members such as CCL7 (MCP3) and CCL8 (MCP2) in OA, and diabetic fracture healing.

Several in vitro and in vivo studies have shown that inflammation is the most important pathogenetic mechanism causing diabetes health complications [31,32]. Although recent studies have evaluated changes and pathways related to some of the MCP family members during diabetic conditions and fracture healing [2,33], much less is known on how hyperglycemic conditions influence the expression and the actions of chemokines in specific skeletal cell types. In this study, we focused on chondrocytes for evaluating the effects of HG on chemokine expression since chondrocytes play a major role in the regulation of fracture healing process and osteoarthritis development [3,34]. Based on our findings that changes in expression levels of *Mcp1*, *Mcp2*, and *Mcp3* in response to HG correlated with the expression levels of matrix metalloproteinase 13 (*Mmp13*) and Osteocalcin (*Oc*), we next determined the role of these chemokines in mediating the effects of HG in chondrocytes by inhibiting the synthesis of chemokines through treatment with bindarit, which has been identified as a specific inhibitor of MCPs [35].

## 2. Materials and Methods

### 2.1. Cell Culture

ATDC5 cell line (Sigma Aldrich, St. Louis, MO, USA), derived from mouse teratocarcinoma fibroblastic cells [36], is characterized as a chondrogenic cell line. ATDC5 cell line is an established and commonly used model for in vitro chondrocyte research [36]. ATDC5 cells were grown in normal growth medium, consisting of DMEM/F12 culture medium (Gibco/Life Technologies, Carlsbad, CA, USA) supplemented with 5% fetal bovine serum (FBS, Life Technology, Carlsbad, CA, USA), 100 Unit/mL penicillin, 100 µg/mL streptomycin (Gibco by Life Technology), in a CO_2_ incubator (5% CO_2_, 95% air) at 37 °C. 10^4^ cells/well were plated in 6-well culture plates in normal growth medium for 48 h prior treatment. To test the effect of HG on chemokine expression, cells were then incubated in: (1) normal growth medium containing 17.5 mM glucose; (2) normal medium supplemented with glucose (Sigma Aldrich, St. Louis, MO, USA) to a final concentration of 55 mM (HG). Then, cells were harvested at three time points. The choice of 55 mM glucose concentration for HG media was based on previous studies using different cell lines [31].

To evaluate the effects of bindarit on chondrocyte cells under HG conditions, after 2 days in normal glucose medium, the cells were subdivided into three groups; cells were treated with (1) normal growth medium containing 17.5 mM glucose; (2) normal growth medium supplemented with glucose to a final concentration of 55 mM (HG); (3) normal growth medium supplemented with HG and 40 µM bindarit (HG+ B). Then, the cells were harvested at day-five of treatment. Bindarit was purchased from MedChem Express (MCE, Monmouth Junction, NJ, USA).

To determine the effect of ascorbic acid (AA) in HG conditions, we treated the cells with AA without adding insulin that can interfere with the effect of HG. Briefly, ATDC5 cells were first plated, in DMEM/F12 medium supplemented with 5% FBS, at 10^4^ cells/well using 6-well-plates. The cells were maintained in normal glucose medium for 48 h. Then, cells were treated as follows: (1) a control group where the cells were maintained in normal glucose medium (Cnt); (2) an AA-normal glucose group, where cells were treated with 50 µg/mL AA and 10 mM β-glycero-phosphate in normal glucose medium; (3) an AA-HG group in which the cells were treated with 50 µg/mL AA, 10 mM βGP and 55 mM final glucose concentration in normal growth medium; (4) an AA + HG+bindarit group in which the cells were treated with 50 µg/mL AA, 10 mM βGP, 55 mM glucose and 40 µM bindarit.

### 2.2. mRNA Quantification: Quantitative Polymerase Chain Reaction (qPCR) for Gene Expression Evaluation

RNA was isolated from ATDC5 cells using RNeasy kit (Qiagen Sciences, LLC, Germantown, MD, USA) and following instructions from the manufacturer protocol.

Reverse transcription was performed with MMLV Reverse Transcriptase (Promega, San Luis Obispo, CA, USA). qPCR was performed using the SYBRGreen master mix (Applied Biosystems, Foster City, CA, USA) and pre-designed primers for tested genes (Integrated DNA Technologies, Coralville, IA, USA). Changes in gene expression were determined as previously described [37]. Briefly, relative differences in expression between groups were determined by qPCR using cycle time (Ct) values as follows: ΔCt values were determined by: Ct value of target gene—Ct value of housekeeping gene from each sample. ΔΔCt values were calculated by subtracting average ΔCt values of control samples from ΔCt value of each treated sample. Then, the relative change in each treated sample was calculated and expressed as relative increase or decrease (fold-change) using 2^−ΔΔCt^. Peptidylprolyl Isomerase A (*Ppia*) was used as internal control gene, and 18S was used to confirm significant differences between groups, so only differences that were found significant using both *Ppia* and 18S control genes were considered.

### 2.3. Statistical Analysis

Four replicates were used per treatment condition, and experiments were repeated two to three times. The results are presented as the means ± standard error of mean (SEM) and were analyzed using one-way ANOVA and Tukey HSD pair-comparisons. Pearson correlation coefficient was calculated using delta Ct values from samples collected at day five-treatment. Significance was adopted for values of *p* ≤ 0.05.

## 3. Results

### 3.1. Effect of HG on the Expression Levels of Different Chemokines and Markers of Chondrogenesis

**Figure 1:**
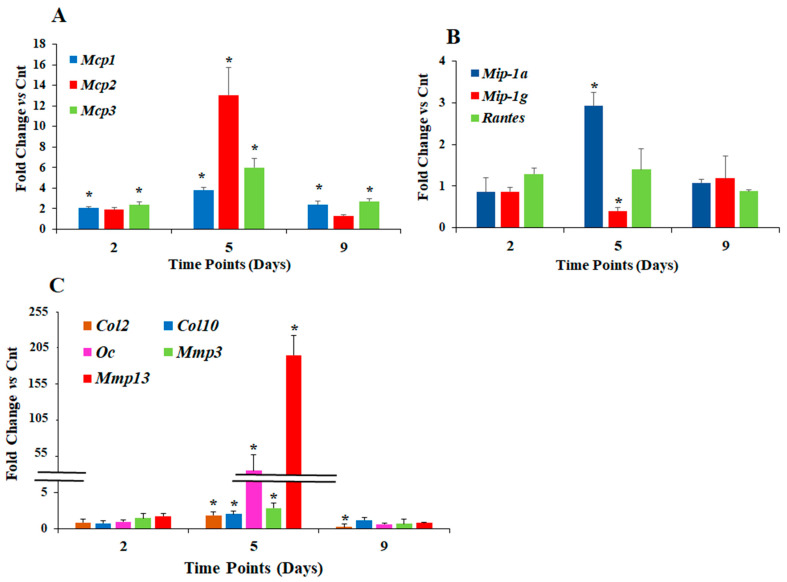
mRNA Expression pattern of different genes at three time points in response to hyperglycemic conditions. *Mcp1*, *2* and *3* (**A**); *Mip-1a*, *Mip-1g* and *Rantes* (**B**). *Col2*, *Col10, Oc*, *Mmp3* and *13* (**C**). ATDC5 cells were cultured in cell culture media with 17.5 mM glucose (Cnt) or 55 mM glucose (HG). Cells were harvested at 3 different time points, and mRNA expression levels were evaluated by qPCR using pre-designed primers for the genes of interest. Data are expressed as fold-change in the expression of each gene in HG treated group compared to Cnt group and are presented as mean ± SEM, *n* = 4, * *p* < 0.05.

HG treatment increased mRNA levels of *Mcp1/Ccl2* at all three time points examined (Figure 1A) with a maximal increase of 4-fold seen at day five (*p* < 0.01) compared to corresponding normal glucose treated cells (Figure 1A). While changes in *Mcp2/Ccl8* mRNA levels in response to HG were not significant at days two and nine, there was a 13-fold rise (*p* < 0.01) in *Mcp2* expression level at day five in HG medium compared to control medium (Figure 1A). *Mcp3/Ccl7* mRNA levels were increased by 2.4-fold at day two, 6-fold at day five, and 3-fold at day nine, in cells treated with HG compared to control (Cnt) normal glucose treated cells (Figure 1A).

To determine whether the increase in mRNA levels of *Mcps* was translated by increased protein levels under HG conditions, we isolated proteins from ATDC5 cells treated with HG and cells treated in normo-glycemic conditions for five days. We then performed dot-blot as described in supplemental materials, a semi-quantitative method to estimate the changes in the expression of specific proteins in HG treated group compared to normal glucose treated group. Dot blot assay showed increased MCP expression in HG treated group compared control group (Figure S1).

While mRNA levels of *Mip-1alpha(a)/Ccl3* and *Mip-1gamma*(g)/*Ccl9* did not change in HG medium at day two, compared to control group (Figure 1B), there was a 3-fold increase in *Mip-1a* expression in HG treated group compared to normal glucose group at day five (Figure 1B, *p* = 0.04), and no change in *Mip-1a* expression was observed at day nine (*p* = 0.7). *Mip-1g* expression was down regulated in HG treated group compared to control group at day five (*p* = 0.045), but its mRNA level returned to normal at day nine. *Rantes* mRNA level was slightly but not significantly upregulated at days two and five in HG treated groups (Figure 1B, *p* > 0.05) and no change was observed in *Rantes* mRNA level at day nine (Figure 1B, *p* = 0.6).

To determine if HG treatment can affect differentiation process of ATDC5 chondrocytes, we evaluated the expression levels of collagen type 2 alpha 1 (*Col2*), collagen type X alpha 1 (*Col10*), *Oc*, and the two major matrix metalloproteinases (*Mmps*) 3 and 13. While HG treatment did not affect the expression levels of these genes during the first two days (Figure 1C), their mRNA levels were significantly increased compared to control group at day-five (*p* < 0.01, Figure 1C). The greatest effect of HG was observed on the expression of *Oc* and *Mmp13* that showed 53- and 200-fold rise in their mRNA levels at day five in HG treated group compared to control group, respectively. However, at day nine of HG treatment, *Col2* mRNA level was significantly downregulated (*p* = 0.007) and mRNA levels of all other genes returned to normal (Figure 1C).

**Figure 2:**
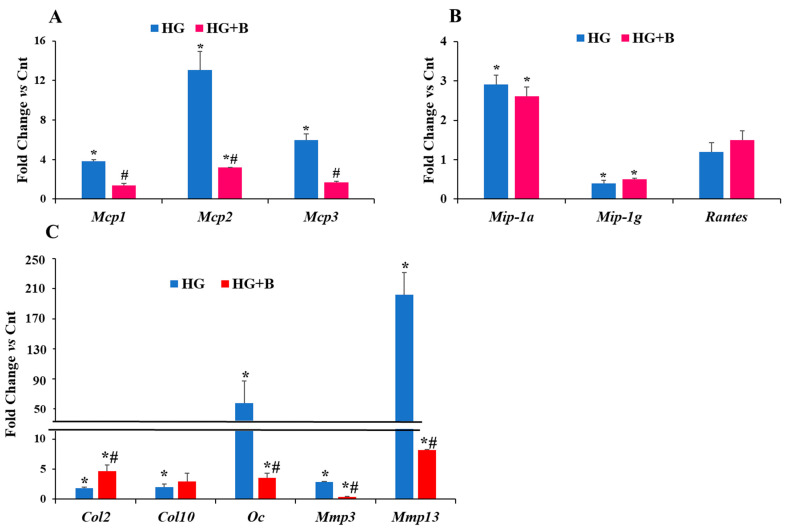
Effect of bindarit on mRNA expression levels of different chemokines, chondrogenesis markers and matrix degrading enzymes after 5 days in HG conditions. *Mcp1*, *2* and *3* (**A**); *Mip-1a*, *Mip-1g* and *Rantes* (**B**). *Col2*, *Col10*, *Oc* and *Mmp3* and *13* (**C**). ATDC5 cell were cultured in cell culture media with normal glucose concentration (Cnt) or 55 mM glucose (HG) in presence (HG+B) or in absence of bindarit. Cells were harvested at day 5. Data are expressed as fold change in mRNA expression of each gene in HG treated group or HG+B group compared to Cnt group, and are presented as mean ± SEM, *n* = 4, * *p* ≤ 0.05 vs. Cnt group, # *p* ≤ 0.05 *vs* HG group.

To determine whether the dramatic effect of HG on *Oc* and *Mmp13* expression at day five is in part mediated via *MCPs*, chondrocyte cells cultured in HG conditions were treated with bindarit, which is one of the specific inhibitors of MCPs [36]. As expected, treatment with bindarit significantly reduced (2- to 6-fold) the expression levels of all three *Mcps* in HG media (Figure 2A). Furthermore, while mRNA level of *Mip-1a* was increased by 3-fold in HG treated group compared to normal glucose group (Figure 2B), *Mip-1g* mRNA level was downregulated in HG medium, *Rantes* was not affected by HG treatment, and bindarit treatment did not change their expression levels in HG medium (Figure 2B). While *Mmp3* and *Mmp13* mRNA levels were downregulated by 2- and 24-fold (Figure 2C), respectively, the expression level of *Col2* was increased by 2.6-fold (*p* < 0.01) in cells treated with bindarit compared to vehicle treatment under HG conditions (Figure 2C). *Col10* expression was upregulated in HG conditions at day five, but bindarit treatment had no significant effect on *Col10*. *Oc* mRNA level was upregulated by 53-fold (*p* < 0.01) in HG treated group (Figure 2C), but this strong effect of HG was reduced by 15-fold in bindarit treated group (*p* < 0.05) compared to the vehicle treated group (Figure 2C).

Table 1 and Table 2 show strong positive correlations between the expression levels of the three *Mcps* (R = 0.88–0.91, *p* < 0.00001, Table 1 and Table 2) as well as with that of *Mmp13* (R = 0.83–0.97, *p* < 0.0001, Table 1 and Table 2). However, *Mip-1g* showed moderate negative correlations with *Col2* (R = −0.67, *p* = 0.03), *Mcp2* (R = −0.56, *p* = 0.05; Table 1 and Table 2), and with *Mmp13* (R = −0.67, *p* = 0.03; Table 1 and Table 2). Strong positive correlations were found between the expression of *Oc* and the three *Mcps* as well as *Mmp13* (R > 0.8, *p* < 0.001; Table 1 and Table 2).

### 3.2. Effect of Ascorbic Acid on the Expression of Different Chemokines and Chondrogenesis Markers in Hyperglycemic Conditions

**Figure 3:**
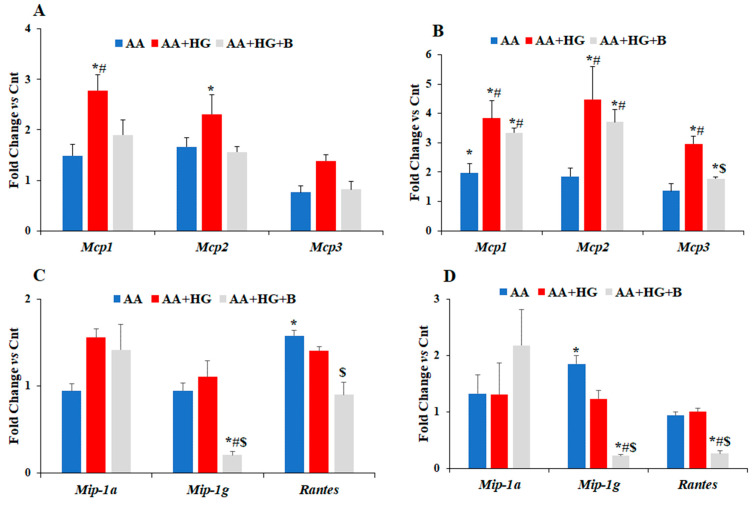
Effect of AA on mRNA expression levels of different chemokines in normo- and hyperglycemic conditions, in presence and in the absence of bindarit. ADTC5 cells were split onto four groups; (1) Control (Cnt) group with normal glucose medium (17.5 mM); (2) AA group with normal glucose medium supplemented with 10 mM beta-glycero-phosphate and 50 µg AA; (3) AA + HG group, cells were cultured in HG medium (55 mM) supplemented with beta-glycero-phosphate and AA; (4) AA and 40 µM bindarit in HG medium (AA + HG + B). The cells were incubated at 37 °C, for three days (**A**,**C**) and five days (**B**,**D**). mRNA expression levels were evaluated by qPCR using pre-designed primers. Data are expressed as fold-change in the expression of each gene in treated group compared to Cnt group and are presented as mean ± SEM. *n* = 3–4, * *p* ≤ 0.05 vs. Cnt group, # *p* ≤ 0.05 vs. AA group, and $ *p* < 0.05 vs. AA + HG.

**Figure 4:**
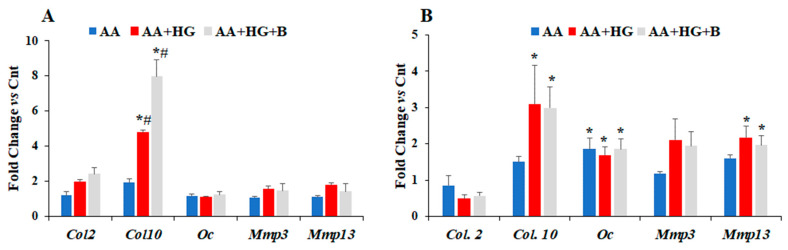
Quantification of the changes in mRNA expression levels of chondrogenesis markers, *Col2*, *Col10* and matrix degrading enzymes in response to AA, in normal and hyperglycemic conditions, in presence and in the absence of bindarit. ADTC5 cells were split into four groups; (1) Control group with normal glucose (17.5 mM) medium; (2) AA group with normal glucose medium supplemented with 10 mM beta-glycero-phosphate and 50 µg AA; (3) AA + HG group, cells were treated with beta-glycero-phosphate and AA in HG conditions (55 mM); (4) AA and 40 µM bindarit in HG conditions (AA + HG + B). The cells were incubated at 37 °C for three (**A**) and five days (**B**). Data are presented as fold change of each gene’s mRNA expression level in AA, AA + HG or AA + HG + B compared to control (Cnt) normal conditions, and are expressed as mean ± SEM, *n* = 4, * *p* ≤ 0.05 vs. Cnt and # *p* ≤ 0.05 vs. AA.

To determine if hyperglycemia affects AA-induced chondrocyte differentiation or vice versa, and, if this effect is mediated via increased expression of *Mcps*, ATDC5 cells were treated with AA in normal, and in HG media, in the presence or absence of bindarit. Then, cells were harvested after three and five day-treatment (Figure 3 and Figure 4). At day three, *Mcp1* and *Mcp2* mRNA levels were increased by 2.8- and 2.3-fold, respectively, in cells treated with AA in HG conditions, and bindarit treatment reversed the effect of HG on *Mcp1* and *Mcp2* mRNA levels in AA and HG treated cells (Figure 3A). However, *Mcp3* mRNA level was unaffected in the cells treated with AA in HG conditions and bindarit treatment had no effect on *Mcp3* expression level in these conditions.

At day five, a 2-fold increase in mRNA level of *Mcp1* (Figure 3B) was observed after treatment with AA in normoglycemic medium, compared to control group. In HG medium its mRNA level increased further up to 4-fold compared to control group (Figure 3B). *Mcp2* and *3* were not affected by treatment with AA in normal glucose medium. However, *Mcp2* and *3* mRNA levels were increased by 3.8 and 4.5-fold, respectively, after five-day treatment with AA in HG conditions. Bindarit treatment partially reduced HG-induced increase in the expression of all three *Mcps* (Figure 3B). The effect of HG on the expression of *Mcp2* was much lower in presence of AA (Figure 3B) than in absence of AA (Figure 2A).

AA treatment did not significantly affect expression of *Mip-1a or Mip-1g* after three-day treatment whether in normal or HG media. However, AA treatment significantly increased *Rantes* mRNA level in normal glucose medium. Furthermore, while bindarit treatment had no effect on the expression of *Mip-1a*, it significantly reduced *Mip-1g* and *Rantes* mRNA levels in AA treated cells under HG conditions (Figure 3C). After five-day treatment with AA, a 2-fold increase in *Mip-1g* mRNA level was observed in normal glucose medium (Figure 3D). However, in HG conditions, the expression of *Mip-1a*, *Mip-1g*, and *Rantes* did not change in AA treated group compared to control group (Figure 3D), and a treatment with bindarit caused a significant reduction in *Mip-1g* and *Rantes* expression compared to AA treated groups in both normal and HG media (Figure 3D).

The effect of HG on the expression levels of chondrogenesis markers was also evaluated in presence of AA. At day three, AA treatment in HG medium increased *Col10* mRNA level that was further upregulated by bindarit treatment (Figure 4A). Expression levels of none of the other genes were affected by AA treatment or bindarit treatment at this time point. However, at day five, *Col10* mRNA level remained significantly greater in HG medium supplemented with AA compared to control normal glucose. *Oc* mRNA level was upregulated in both normal and HG media after five-day treatment with AA (Figure 4B), and the expression level of *Mmp13* was increased in AA treated cells under HG conditions, but bindarit treatment under HG conditions had no effect on the expression levels of any of the chondrogenesis markers tested after five-day in the presence of AA (Figure 4B).

## 4. Discussion

Chemokines are known for their role on leukocyte recruitment and osteoclast proliferation and differentiation and are thought to play important roles in the regulation of bone metabolism [38,39]. They may contribute to the regulation of bone repair by integrating the inflammatory events and the reparative mechanisms important in modulating fracture healing. Previous studies have reported the involvement of some chemokines in bone remodeling and fracture repair [14], with MCP-1 being the most studied in bone, osteoarthritis, and diabetes fields [2,14,16,26]. Furthermore, fracture healing in diabetic patients showed several complications such as delayed healing, non-union and pseudoarthrosis [40,41] but the cell types that contribute to impaired healing under hyperglycemic conditions are not well established. The process of fracture healing involves initial inflammation followed by chondrogenesis, endochondral ossification and remodeling of the fracture callus bone. Since chondrogenesis represents an important step that contributes to overall fracture healing, we tested in this study the effect of hyperglycemic conditions on the expression of different chemokines in chondrocyte cells. We evaluated the response to hyperglycemia of three *Mcps* and other chemokines that are differentially regulated in response to bone fracture [14] and are known to regulate trafficking of immune cells [42,43].

In the present study, we evaluated the effect of HG conditions on the expression of three *Mcps* as well as *Mmps* and other chondrogenesis markers in chondrocyte cells. We also tested the effect of blocking synthesis of MCPs, on the expression of *Mmps* and chondrogenesis markers in chondrocyte cells, under HG conditions. The results of this study showed that five-day treatment of chondrocyte cells with 55 mM glucose (to mimic hyperglycemic conditions) up regulated the expression of the three *Mcps* as well as the expression of *Mmp3*, *Mmp13* and *Oc*. The increase in *Mcp1*and *Mcp2* expression levels after five-day culture under HG conditions, was confirmed by immuno-dot blot using proteins isolated from chondrocyte cells (Figure S1, Supplementary Materials). Furthermore, strong positive correlations were found between mRNA expression levels of the three *Mcps* and *Mmp13*, suggesting that hyperglycemia up-regulates the expression of *Mcps* which in turn induces the expression of *Mmp13* that is known to play major roles in cartilage degeneration and differentiation [44]. These data together suggest a potential role of MCPs in the regulation of *Mmp13* expression, but this hypothesis still needs to be confirmed with more investigations. Recent studies have demonstrated that MCP2 activates NF-κB signaling pathway and promotes the migration and invasion of esophageal squamous carcinoma cells [45]. In the present study, among the three MCPs investigated in chondrocyte cells, MCP2 was the most affected by HG, but this effect faded with time as was revealed by a reduction in expression of *Mcp2* after nine days in HG conditions. These data suggest a potential role of MCP2 in regulation of *Mmp13* expression in chondrocytes, and its catabolic effect is maintained through activation of NF-κB signaling pathways in presence of inflammatory cells. However, more investigations are needed to confirm this hypothesis. Osteocalcin, which is a marker of mature osteoblasts and hypertrophic cartilage, was upregulated in HG conditions, its mRNA level correlated with the expression of both *Mmps* and the three *Mcps*, which suggests that HG conditions can lead to premature chondrocyte trans-differentiation through increased expression of *Mcps* and *Mmps*.

Bindarit, the small molecule that specifically inhibits the synthesis of MCPs [35], reversed the effect of hyperglycemia on the expression of *Oc* and *Mmp13*. Bindarit, at the smallest dose previously tested with other cell lines [46], not only alleviated hyperglycemia-induced inflammation but showed an anabolic effect since treatment of chondrocytes with bindarit in hyperglycemic conditions upregulated the expression of *Col2*, which is one the major markers of chondrocyte proliferation. Since chondrogenesis plays an important role in fracture healing, these findings raise the possibility that bindarit is a target molecule to treat fracture healing complications in diabetic patients.

Ascorbic acid, also known as vitamin C, plays a central role in chondrocyte differentiation besides its antioxidant effects [47,48]. Vitamin C has been shown to promote hypertrophic differentiation of cultured chick embryo chondrocytes as well as ATDC5 chondrocytes [49,50,51,52]. In the present study, we also found that treatment with AA, as previously described [50], reduced the effect of hyperglycemia on the expression of *Mcps* and *Mmp13* in chondrocyte cells, probably by reducing oxidative stress (which is one of the main causes for MCP1 secretion) [53].

The three MCPs tested in this study are known for their role in chemotaxis of inflammatory cells. They are differentially expressed during early phase of post fracture healing in normoglycemic animals [22,37]. However, extended expression of the three *Mcps* beyond the first week-post fracture was reported in animal models of delayed fracture healing such as in diabetic animals [54]. MCPs have also been found to be upregulated in human and murine osteoarthritic knees [4]. In addition, Bindarit was successfully used to mitigate cartilage damage, synovitis, osteophyte formation and macrophage accumulation in mouse injured knees [4]. In the present study, our data showed for the first time that MCPs play an important role in initiating chondrocyte degeneration independently of inflammatory cells under hyperglycemic conditions.

Major evidence from both basic scientific research and clinical studies have shown that diabetes is associated with delayed or impaired fracture healing [55]. Alblowi et al., [54] reported significant increase in mRNA level of *Mcp2* among other chemokines in fracture calluses of diabetic mice compared to normoglycemic mice at the transition phase from cartilage callus to hard callus [54] suggesting that MCP2 is involved in premature cartilage loss observed in diabetic fracture healing [56].

Acute inflammation is crucial for fracture healing and for the faith of callus cartilage in fracture healing, but uncontrolled inflammation can lead to delayed fracture healing or non-union. Based on the above findings, we postulate that prolonged expression of *Mcps* under hyperglycemia can induce *Mmps* expression and promote cartilage degradation and poor healing. Thus, MCPs’ inhibitors such as bindarit could be a target for future investigations to improve diabetic fracture healing.

## 5. Conclusions

In this study, we have shown that hyperglycemia upregulates the expression of monocyte chemotactic protein family and matrix degrading enzymes in chondrocyte cells. Furthermore, treatment with bindarit, known to specifically inhibit synthesis of MCPs, blunts the effect of HG in chondrocyte cells by relieving inflammation-induced by hyperglycemia.

## Figures and Tables

**Table 1:** Pearson correlation coefficient from delta Ct values of samples collected after five-day treatment.

R Values	*Col2*	*Col10*	*Oc*	*Mmp3*	*Mmp13*	*Mcp1/ccl2*	*Mcp2/Ccl8*	*Mcp3/Ccl7*	*Mip-1a*	*Mip-1g*	*Rantes*
*Col2*	1	0.27	−0.67	−0.06	−0.62	−0.39	−0.65	−0.62	0.60	−0.67	0.42
*Col10*		1	−0.38	−0.21	−0.20	−0.44	−0.25	−0.47	−0.27	−0.23	−0.12
*Oc*			1	0.78	0.98	0.88	0.98	0.93	−0.40	−0.50	−0.45
*Mmp3*				1	−0.48	0.62	0.61	0.65	0.70	−0.50	−0.16
*Mmp13*					1	0.83	0.97	0.87	0.60	−0.67	−0.27
*Mcp-1*						1	0.88	0.91	0.69	−0.4	−0.35
*Mcp-2*							1	0.91	0.70	−0.56	−0.26
*Mcp-3*								1	0.55	−0.24	−0.28
*Mip-1a*									1	−0.60	0.15
*Mip-1g*										1	0.25

**Table 2:** P values of Pearson correlation from delta Ct values of samples collected after five day treatment.

*p* Values	*Col2*	*Col10*	*Oc*	*Mmp3*	*Mmp13*	*Mcp1/Ccl2*	*Mcp2/Ccl8*	*Mcp3/Ccl7*	*Mip-1a*	*Mip-1g*	*Rantes*
*Col2*	<0.01	0.39	0.03	0.85	0.032	0.34	0.08	0.10	0.04	0.03	0.15
*Col10*		<0.01	0.23	0.51	0.52	0.15	0.44	0.12	0.31	0.12	0.22
*Oc*			<0.01	0.01	<0.01	<0.01	<0.01	<0.01	0.11	0.11	0.70
*Mmp3*				<0.01	0.11	0.04	0.04	0.04	0.03	0.06	0.12
*Mmp13*					<0.01	<0.01	<0.01	<0.01	0.04	0.03	0.63
*Mcp-1*						<0.01	<0.01	<0.01	0.03	0.12	0.31
*Mcp-2*							<0.01	<0.01	0.04	0.05	0.32
*Mcp-3*								<0.01	0.05	0.06	0.30
*Mip-1a*									<0.01	0.07	0.31
*Mip-1g*										<0.01	0.50

## Supplementary Materials

The following supporting information can be downloaded at: https://www.mdpi.com/article/10.3390/life12060836/s1, Figure S1: Dot blot assay showed increased MCP1 and MCP2 expression in chondrocyte cells under high glucose (HG) conditions compared to control (Cnt) normal glucose medium.
