# Supplementary material for: Monocyte Chemotactic Proteins Mediate the Effects of Hyperglycemia in Chondrocytes: In Vitro Studies
Source: Life (Basel). 2022 Jun 3;12(6):836. doi: 10.3390/life12060836 (PMC9224901; doi:10.3390/life12060836)
Supplement: Supplementary file 1 [file life-12-00836-s001.zip › life-1712161-supplementary.pdf]

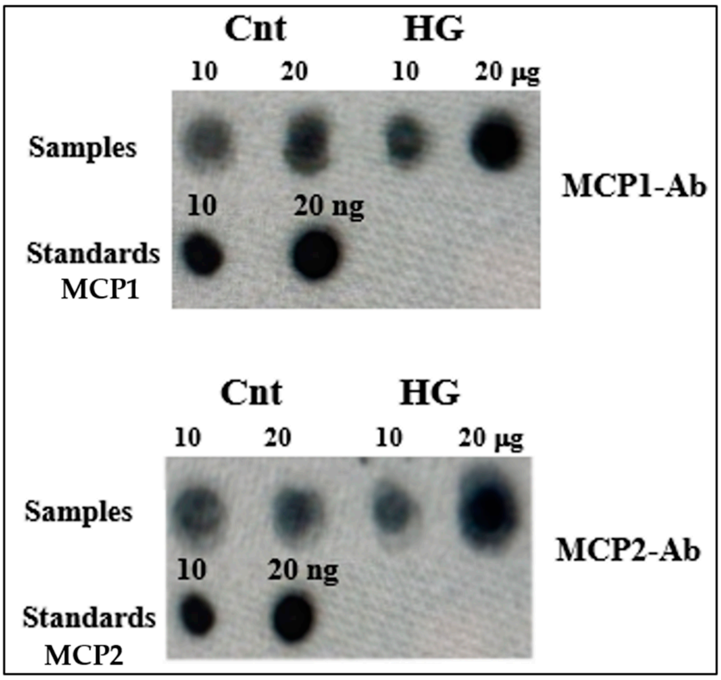

Figure S1. Dot blot assay showed increased MCP1 and MCP2 expression in chondrocyte cells under high glucose (HG) conditions compared to control (Cnt) normal glucose medium. ATDC5 cells were seeded in 6-well culture plates at a density of 10 000 cell/well, in normal growth medium that consists of DMEMF12 supplemented with 5% FBS, as described in Materials and Methods. After 2 days, media was changed, and group 1 cells were incubated in normal growth media with 17.5 mM glucose (Cnt), and group 2 cells were incubated in normal growth media supplemented with glucose to a final concentration of 55 mM (HG). 3-wells from the 6-well plate were used for each treatment. Cells were harvested at day-5 using radioimmunoprecipitation assay buffer (RIPA buffer) supplemented with Protease inhibitors cocktail (Abcam). 10 and 20 ug protein from each treatment were loaded in Nitrocellulose membrane. 10 and 20 ng standard murine recombinant proteins MCP1 and MCP2 (PeproTech, East Windsor, NJ, USA) were used as positive controls. Non-specific sites were blocked by soaking the membrane in 5% bovine serum albumin (BSA). Then, the membranes were incubated, for 2 h at room temperature, in rabbit polyclonal MCP1-antibody (Ab, PeproTech), at 0.5 μg/mL and MCP2-Ab (ThermoFisher Scientific, Waltham, MA, USA) at 2.5 μg/mL. Membranes were incubated in goat anti-rabbit HRP conjugate for 30 min. Then, membranes were incubated, for 2 min, in enhanced chemiluminescent (ECL) substrate to detect HRP. Images were taken using Azure Biosystems (Azure Biosystems, Dublin, CA, USA).
